# The Potential Effects on Microbiota and Silage Fermentation of Alfalfa Under Salt Stress

**DOI:** 10.3389/fmicb.2021.688695

**Published:** 2021-10-11

**Authors:** Qiang Lu, Zhen Wang, Duowen Sa, Meiling Hou, Gentu Ge, ZhiJun Wang, Yushan Jia

**Affiliations:** ^1^College of Grassland and Resources and Environment, Inner Mongolia Agricultural University, Hohhot, China; ^2^Grassland Research Institute, Chinese Academy of Agricultural Sciences, Hohhot, China; ^3^College of Agriculture, Inner Mongolia University for Nationalities, Tongliao, China

**Keywords:** alfalfa, salt stress, silage, fermentation quality, microbial community

## Abstract

This study investigated the fermentation quality of alfalfa grown in different salt stress regions in China. Following the production of silage from the natural fermentation of alfalfa, the interplay between the chemical composition, fermentation characteristics, and microbiome was examined to understand the influence of these factors on the fermentation quality of silage. The alfalfa was cultivated under salt stress with the following: (a) soil content of <1%0 (CK); (b) 1–2%0 (LS); (c) 2–3%0 (MS); (d) 3–4%0 (HS). The pH of the silage was high (4.9–5.3), and lactic acid content was high (26.3–51.0 g/kg DM). As the salt stress increases, the NA^+^ of the silages was higher (2.2–5.4 g/kg DM). The bacterial alpha diversities of the alfalfa silages were distinct. There was a predominance of desirable genera including *Lactococcus* and *Lactobacillus* in silage produced from alfalfa under salt stress, and this led to better fermentation quality. The chemical composition and fermentation characteristics of the silage were closely correlated with the composition of the bacterial community. Furthermore, NA^+^ was found to significantly influence the microbiome of the silage. The results confirmed that salt stress has a great impact on the quality and bacterial community of fresh alfalfa and silage. The salt stress and plant ions were thus most responsible for their different fermentation modes in alfalfa silage. The results of the study indicate that exogenous epiphytic microbiota of alfalfa under salt stress could be used as a potential bioresource to improve the fermentation quality.

## Introduction

Alfalfa (*Medicago sativa* L.) is one of the most important forage, with high protein and highly digestible fiber contents and salinization tolerance. Not only is alfalfa showing high salt tolerance to saline-alkali soil, but appropriate salt stress would improve the quality of alfalfa as well, including amino acids, proteins, and other important nutrients (Liu et al., 2019). Soil salinization has become an important factor in constraining global food and forage crops (Lu et al., 2021). According to statistics from UNESCO and FAO, soil salinization has a presence in more than 100 countries around the world, with an area of 932.2 Mha (Rengasamy, 2006). Soil salinization is an issue affecting the development of global agriculture and animal husbandry (Xiao et al., 2019).

In the salinization land, alfalfa can not only supply adequate forage resources but also provide a large amount of high-quality protein feed for animal husbandry (Guo et al., 2013). In particular, competition for ionic factors such as K^+^, Na^+^, and Cl^−^ would lead to plant nutrient imbalance (Mahmoud et al., 2017). It has been documented that excessive concentrations of Na^+^ in plant tissues prevent nutrient and osmotic balance, leading to specific ionic toxicity (Hariadi et al., 2010). Moreover, from the perspective of plant chemical composition metabolism, with the increase of salt stress, the content of proline in crops also gradually increased (Sanada et al., 1995). In the study by Xue et al. (2018) tomato's soluble sugar and soluble protein eventually got an increasing trend in response to salt stress. Soil salinization forage is a potential and huge source of forage. The demand for alfalfa is large, but production is affected by seasonal harvest. In order to combat this and meet the year-round demand, alfalfa needs proper pretreatment and conservation.

Ensiling has been recognized as a good way of keeping forage nutrition, and as this method preserves protein, it improves forage palatability for livestock as well (McDonald et al., 1991). The quality of silage mainly depends on LAB (lactic acid bacteria). LAB could inhibit not only the growth of spoilage bacteria but also WSC (catabolize water-soluble carbohydrates) to lactic acid and decrease pH (Li et al., 2018). Lower pH would inhibit spoilage microorganisms, including primary members of *Enterobacteriaceae*, clostridial spores, yeast, and molds (Bolsen et al., 1996). From a silage preservation perspective, the loss of dry matter (DM) is also critical. Therefore, making use of microbial resources to carry out fermentation and consume the least energy to preserve nutrients is very important.

However, the biotechnological potential of LAB involved in silage fermentation remains poorly explored. To seek the better fermentation effect as we provided, we need to excavate the microbial resources in different habitats to obtain LAB. Indigenous strains could be isolated from the habitat in which they will be inoculated, which can confer competitive advantages because the efficacy of silage inoculant bacteria is based on their ability to compete effectively with the epiphytic microbiota in forages (Duffner and O'Connell, 1995; Fabiszewska et al., 2019). Besides, the microbial diversity in silages has mainly been studied using culture-independent techniques [polymerase chain reaction (PCR)-based techniques], revealing a wide diversity of unidentified microorganisms in the fermentation (McAllister et al., 2018). To the best of our knowledge, few researchers have investigated the potential of alfalfa nutrition and microbiota under salt stress. Thus, the aim of this study was to investigate the potential of alfalfa microbiota under salt stress and the effects of ions on silage fermentation characteristics and microbial community, supplying the theoretical basis for controlling silage fermentation.

## Materials and Methods

### Site Description

The experiment was conducted at the Baotou Experimental Station for Forage Processing and High Efficient Utilization, Inner Mongolia Agricultural University. The experimental station was located in Baotou City in Inner Mongolia, China, which was a typical area with high salinity in the Hetao Plain. The land spans between 110°37″ −110°27″ E and 40°05″ −40°17″ N. The climate is a north temperate continental climate with arid and windy conditions. The dominant wind direction throughout the year is northwest wind. The annual average temperature is 6.8°C, and the frost-free period is about 165 days. The annual average rainfall is 330 mm, and the annual average evaporation is 2,094 mm.

### Description of Raw Materials

The field experiments were conducted in 2019, and the variety of alfalfa tested was the ZhongMu No.3, provided by the Beijing Institute of Animal Science and Veterinary Medicine of the Chinese Academy of Agricultural Sciences. The characteristics of alfalfa were strong salt resistance, good palatability, high nutrition, and rich in value. Alfalfa was sown in May 2018, the sowing method was a drill, and the row-to-row distance was 10 cm. Four positions were selected to represent various salt stress levels, i.e., non-salt stress (CK), light salt stress (LS), moderate stress (MS), and severe stress (HS). The salt stress contents in CK, LS, MS, and HS sites were <1, 1–2, 2–3, and 3–4%0, respectively. Each treatment was repeated three times. The physical properties of the soil are shown in Table 1.

**Table 1:** The physical and chemical properties of soils.

**Indictors**	**Na^**+**^(g/kg)**	**K^**+**^(g/kg)**	**pH**	**EC (mS/cm)**
CK	0.11 ± 0.006c	0.027 ± 0.001d	7.4 ± 0.21b	0.21 ± 0.02a
LS	0.15 ± 0.004b	0.031 ± 0.001c	8.4 ± 0.08a	0.59 ± 0.10b
MS	0.16 ± 0.002b	0.035 ± 0.001b	8.6 ± 0.11a	1.35 ± 0.01c
HS	0.25 ± 0.019a	0.041 ± 0.001a	8.7 ± 0.34a	2.3 ± 0.29d

*CK, without salt stress; LS, under light salt stress; MS, under moderate salt stress; HS, under severe salt stress; EC, electrical conductivity. Numbers in a column followed by different lowercase letters differ at p <0.05*.

Alfalfa was harvested in the initial flowering stage, then wilted for 5 h to obtain a targeted DM content, and immediately chopped into 2- to 3-cm lengths by a fodder chopper. Each material was treated separately to prevent crossing contaminations. Two kilograms of the prepared alfalfa were packed in polyethylene plastic bags and sealed with a vacuum sealer in each group. All bags were assigned without additives. Triplicates for each treatment were opened after 30 and 60 days of ensiling, respectively.

### Chemical Composition and Organic Acid

The DM of the fresh alfalfa and silage was determined by oven drying at 65°C for 72 h. The neutral detergent fiber (NDF) and acid detergent fiber (ADF) were measured according to Van Soest's procedures (Van et al., 1991). Colorimetry after reaction with anthrone reagent was used to determine the starch and water-soluble carbohydrate (WSC) content (Lei et al., 2015). Non-structural carbohydrates (NSC) are the sum of WSC and starch. The crude protein (CP = total N × 6.25) was determined using a Kjeldahl apparatus (Gerhart Vapodest 50 s, Germany) according to Patrica (1997). The concentrations of Na^+^ and K^+^ ions of alfalfa were measured relative to standard solutions using a model 425 flame photometer (Sherwood Scientific Ltd., UK).

To determine the fermentation traits of forage, a sample (10 g) of silage was mixed with 90 g of deionized water kept at a 4°C fridge for 24 h. The liquid extract was filtered through four layers of cheesecloth and filtered paper. The prepared filtrates were determined for measuring pH, ammonia nitrogen (ammonia-N), and organic acids. The pH was measured immediately with a glass electrode pH meter (LEICI pH S-3C, Shanghai, China). The content of ammonia-N was followed by the phenol-hypochlorite procedure of Broderick and Kang (1980). The concentration of organic acids was determined by high-performance liquid chromatography (HPLC, Waters e2695, Massachusetts USA; column: Waters Symmetry C18; oven temperature, 50°C; mobile phase, 3 mmol L^−1^ perchlorate solution; flow rate, 1.0 ml min^−1^; flame photometric detector, 210 nm; sample size, 5.0 μl) of Ping et al. (2017). Buffering capacity (BC) was determined by the hydrochloric acid sodium hydroxide method (Lin et al., 1992).

### Microorganisms Enumeration

For enumeration of the microorganisms, 10 g of fresh sample or silage was shaken with 90 ml of sterile distilled water at 120 rpm for 2 h. Then, 1 ml of solution was used for 10-fold serial dilution for microorganism enumeration and then the remaining solution was filtered and stored in the −80°C refrigerator for DNA extraction. The colonies of LAB (lactic acid bacteria) were counted on MRS agar medium after incubation in an anaerobic incubator (Heal Force Instrument Manufacturing Co., Ltd., Shanghai, China) at 37°C for 48 h. Aerobic bacteria were cultured and counted on nutrient agar medium (Nissui seiyaku Ltd., Tokyo, Japan). Yeasts were counted on potato dextrose agar (Nissui seiyaku Ltd., Tokyo, Japan). *Enterobacteriaceae* was counted on the Violet Red Bile Glucose Agar medium after 48 h of incubation at 37°C under aerobic conditions. The microbial data were obtained as colony-forming units (CFU) and were transformed to a logarithmic scale on a fresh matter (FM) basis.

### High-Throughput Sequencing of Microbial Population

Microbial DNA of alfalfa samples was extracted using the E.Z.N.A.® soil DNA Kit (Omega Bio-Tek, Norcross, GA, U.S.) according to the manufacturer's protocols. The final DNA concentration and purification were determined by NanoDrop 2000 UV-vis spectrophotometer (Thermo Scientific, Wilmington, USA), and DNA quality was checked by 1% agarose gel electrophoresis. The V3–V4 hypervariable regions of the bacteria 16S rRNA gene were amplified with primers 338F (5′-ACTCCTACGGGAGGCAGCAG-3′) and 806R (5′-GGACTACHVGGGTWTCTAAT-3′) by thermocycler PCR system (GeneAmp 9700, ABI, USA). The PCR reactions were conducted using the following program: 3 min of denaturation at 95°C, 27 cycles of 30 s at 95°C, 30 s for annealing at 55°C, and 45 s for elongation at 72°C, and a final extension at 72°C for 10 min. PCR reactions were performed in triplicate 20-μl mixture containing 4 μl of 5 × FastPfu Buffer, 2 μl of 2.5 mM dNTPs, 0.8 μl of each primer (5 μM), 0.4 μl of FastPfu Polymerase, and 10 ng of template DNA. The resulting PCR products were extracted from a 2% agarose gel and further purified using the AxyPrep DNA Gel Extraction Kit (Axygen Biosciences, Union City, CA, USA) and quantified using QuantiFluor™-ST (Promega, USA) according to the manufacturer's protocol.

Raw fastq files were demultiplexed, quality-filtered by Trimmomatic, and merged by FLASH with the following criteria: (a) the reads were truncated at any site receiving an average quality score <20 over a 50-bp sliding window; (b) primers were exactly matched allowing two nucleotide mismatching, and reads containing ambiguous bases were removed; (c) sequences whose overlap was longer than 10 bp were merged according to their overlap.

Operational taxonomic units (OTUs) were clustered with 97% similarity cutoff using UPARSE, and chimeric sequences were identified and removed using UCHIME. The taxonomy of each 16S rRNA gene sequence was analyzed by the RDP Classifier algorithm against the Silva (SSU123) 16S rRNA database using a confidence threshold of 70%.

### Statistical Analysis

The statistical data were analyzed by the procedure of SAS (version 9.3, SAS Institute Inc., Cary, NC, USA). Duncan's multiple range tests were used to evaluate differences among treatments. High-throughput sequencing data were performed using an online platform of Majorbio I-Sanger Cloud Platform (www.i-sanger.com).

## Results

### The Chemical Composition of Fresh Alfalfa

The chemical composition and microbial populations of fresh materials and stressed by salt are described in Table 2. The alfalfa DM content ranged from 300 g/kg to 313 g/kg; the MS and HS samples had the highest and lowest DM contents, respectively (*p* < 0.05). There was no significant difference between the CP, ADF, and NSC (*p* > 0.05). The SP content (108 g/kg) of LS was significantly higher than the others (*p* < 0.05). There was no significant difference among the treatments (*p* > 0.05), but the WSC content (49 g/kg) in CK was significantly higher than in other groups (*p* < 0.05). Also, salt stress significantly affected the Na^+^ and K^+^ content of alfalfa (*p* < 0.05). The K^+^ content in the HS (5.1 g/kg) was significantly highest (*p* < 0.05). Furthermore, It proved that the microbial population on plants could be affected by salt stress. The content of LAB (lactic acid bacteria) in MS (7.7 Log_10_ cfu/g) and HS (7.6 Log_10_ cfu/g) was significantly higher than that in CK (7.1 Log_10_ cfu/g) and LS (6.91 Log_10_ cfu/g).

**Table 2:** Chemical composition and microbial populations of the fresh alfalfa.

**Items**	**CK**	**LS**	**MS**	**HS**	**SEM**	***p*-value**
Dry matter (g/kg FM)	303b	310a	313a	300b	0.02	0.37
Crude protein (g/kg DM)	204a	228a	207a	208a	0.42	0.19
Soluble protein (g/kg DM)	98a	108a	69b	66b	0.56	<0.0001
Acid detergent fiber (g/kg DM)	368a	347a	368a	384a	0.63	0.26
Neutral detergent fiber (g/kg DM)	439bc	419c	463a	459ab	0.61	0.01
Non-structural carbohydrate (g/kg DM)	53a	50a	44a	45a	0.20	0.42
Water-soluble carbohydrate (g/kg DM)	49a	45ab	32c	37bc	0.24	0.02
Buffering capacity (mEq/kg DM)	309c	331b	349ab	359a	6.37	0.01
Na^+^ (g/kg DM)	1.9d	2.7c	4.8b	5.1a	0.40	<0.0001
K^+^ (g/kg DM)	26.73	31.2a	30.9b	29.4c	0.53	0.01
Lactic acid bacteria (Log_10_ cfu/g FM)	7.1b	6.9c	7.7a	7.6a	0.10	0.02
Aerobic bacteria (Log_10_ cfu/g FM)	7.9a	7.2b	7.7a	7.5a	0.09	0.02
Yeast (Log_10_ cfu/g FM)	6.7b	6.4b	7.2a	6.6b	0.12	<0.0001
*Enterobacteriaceae* (Log_10_ cfu/g FM)	7.0a	6.3b	6.8ab	7.3a	0.13	<0.0001

*CK, without salt stress; LS, under light salt stress; MS, under moderate salt stress; HS, under severe salt stress; DM, dry matter; FM, fresh matter; cfu, colony-forming unit; SEM, standard error of means. Means with different letters in the same column (a–d) differ (p <0.05)*.

### Fermentation Characteristics and Chemical Composition of Alfalfa Silage

The fermentation characteristics of alfalfa silage on different days are shown in Table 3. Factor analysis illustrated that salt stress (T), the ensiling day (D), and the interaction of salt stress and the ensiling day had significantly (*p* < 0.05) affected LA (Lactic acid), AA (Acetic acid), BA (Butyric acid), and ammonia-N (Ammonia nitrogen) contents. Salt stress significantly affects the content of LA, AA, BA, and ammonia-N (*p* < 0.0001). After 60 days of ensiling, the pH of the silages ranged from 4.9 to 5.3, and the MS silage achieved significantly lower pH values and higher LA concentration as compared to other silage (*p* < 0.05). With the rapid decrease in pH, the WSC (17 g/kg) content of MS at 60 days was reduced by 15 g/kg compared to 0 days (32 g/kg). The AA of the silages ranged from 31.4 to 45.1; as the salt stress increases, the accumulation of AA presents a trend of increase and then decrease throughout ensiling of four groups, resulting in the highest ratio of highest (*p* < 0.05) AA concentration after 60 days of ensiling in the MS. The BA of the silages ranged from 11.4 to 26.9; the content of BA in CK was significantly highest at 60 days (21.7 g/kg) of fermentation (*p* < 0.05), and the content of LA (31.7 g/kg) was also significantly lowest (*p* < 0.05). After 60 days of silage, the ammonia-N content in the MS and HS group was notably (*p* < 0.05) higher than LS and CK. In this research, the Na^+^ content in MS and HS groups was also significantly (*p* < 0.05) higher than LS and CK.

**Table 3:** Fermentation characteristics of alfalfa silage after 30 and 60 days of ensiling.

**Items**	**Treatment**	**Ensiling (days)**		**Means**	**SEM**	***p*** **-value**		
		**30**	**60**			**T**	**D**	**T × D**
pH	CK	5.1ab	5.3a	5.2a	0.05	0.02	0.02	0.22
	LS	5.0b	5.3a	5.2a				
	MS	4.9b	4.9b	4.8b				
	HS	4.9b	5.2ab	5.0ab				
Lactic acid (g/kg DM)	CK	26.3f	31.7e	28.9d	1.54	<0.0001	0.02	<0.0001
	LS	45.6b	39.5d	42.5b				
	MS	43.2bc	51.0a	47.1a				
	HS	40.1cd	40.2cd	40.2c				
Acetic acid (g/kg DM)	CK	46.7a	45.1ab	45.9a	1.37	<0.0001	0.0005	0.0006
	LS	41.2b	47.9a	44.6a				
	MS	32.9c	32.7c	32.8c				
	HS	31.4c	42.0b	36.7b				
Butyric acid (g/kg DM)	CK	16.4bc	26.9a	21.7a	1.00	<0.0001	<0.0001	<0.0001
	LS	15.4c	17.2b	16.3b				
	MS	12.7d	11.4d	12.1c				
	HS	13.1d	11.5d	12.3c				
Ammonia-N (g/kg DM)	CK	24.3cd	33.6a	28.9a	1.29	<0.0001	0.0002	0.02
	LS	27.3bc	29.5ab	28.4a				
	MS	17.7e	18.4e	18.1b				
	HS	15.4e	22.6d	19.0b				

*CK, without salt stress; LS, under light salt stress; MS, under moderate salt stress; HS, under severe salt stress; FM, fresh matter; T, salt stress; D, ensiling days; T × D, the interaction between salt stress and ensiling days; SEM, standard error of means. Means with different letters in the same column (a–f) differ (p <0.05)*.

The chemical composition of alfalfa silage on different days are shown in Table 4. Factor analysis illustrated that salt stress (T), the ensiling day (D), and the interaction of salt stress and ensiling day had a significant (*p* < 0.05) effect on WSC and NSC contents. Salt stress significantly (*p* < 0.05) affects the content of DM, NSC, SP, and NA^+^. After 60 days of ensiling, the DM losses ranged from 0.7 g/kg to 27.2 g/kg. The WSC content in the four groups was consumed more (43.8%−73.3%) than the NSC content, while the NSC content was consumed between 22.2% and 38.0%. It was the large consumption of WSC that leads to the rapid reproduction of LAB. In contrast, starch was less consumed during ensiling. Factor analysis illustrated that salt stress (T) and ensiling day (D) had a significant (*p* < 0.0001) effect on SP content. Compared with the alfalfa raw materials of each group, the SP content increased by 45.4–121.2%. After 60 days of ensiling, the SP content in LS (157 g/kg) silage was significantly higher than in other groups. At the same time, the content of ammonia-N was also the lowest. This may inhibit the further decomposition of CP by protease and then eventually leads to the accumulation of SP content. After 60 days of ensiling, NA^+^ content (5.1 g/kg) was significantly higher than CK, LS, and MS (*p* < 0.0001). The NA^+^ content changes a little after ensiling.

**Table 4:** Chemical composition of alfalfa silage after 30 and 60 days of ensiling.

**Items**	**Treatment**	**Ensiling (days)**		**Means**	**SEM**	***p*** **-value**		
		**30**	**60**			**T**	**D**	**T × D**
Dry matter (g/kg FM)	CK	296c	303c	300c	1.02	<0.0001	0.87	0.76
	LS	304c	307c	305c				
	MS	285b	284b	285b				
	HS	313a	307a	310a				
Dry matter loss(g/kg FM)	CK	8.4ab	0.9b	8.9b	10.3	0.0017	0.88	0.81
	LS	3.03ab	0.7b	1.9c				
	MS	26.6a	27.2a	26.9a				
	HS	10.1b	3.8b	6.9b				
Water-soluble carbohydrates (g/kg DM)	CK	25a	14cd	19.5a	0.88	0.01	<0.0001	0.01
	LS	17c	12d	14.3b				
	MS	21b	17c	18.8a				
	HS	16c	14cd	15.2b				
Non-structural carbohydrates (g/kg DM)	CK	37b	38b	37.3b	1.09	<0.0001	<0.0001	0.03
	LS	27d	31c	29.2d				
	MS	37b	44a	40.3a				
	HS	29cd	35b	32.3c				
Crude protein (g/kg DM)	CK	195c	211ab	203c	1.84	0.01	0.11	0.05
	LS	217ab	221a	219a				
	MS	213ab	208b	210b				
	HS	209b	211ab	210bc				
Soluble protein (g/kg DM)	CK	133c	148b	140b	2.24	<0.0001	<0.0001	0.45
	LS	150ab	157a	154a				
	MS	124d	136c	130c				
	HS	137c	146b	141b				
K^+^ (g/kg DM)	CK	29.9ab	29.9ab	29.9a	0.38	0.84	0.12	0.28
	LS	29.6ab	30.3ab	29.9a				
	MS	29.2ab	29.7ab	29.4a				
	HS	27.2b	31.1a	29.1a				
Na^+^ (g/kg DM)	CK	2.2c	2.2c	2.2d	0.26	<0.0001	0.11	0.18
	LS	2.6c	2.7c	2.6c				
	MS	4.6b	4.6b	4.6b				
	HS	4.7b	5.4a	5.1a				

*CK, without salt stress; LS, under light salt stress; MS, under moderate salt stress; HS, under severe salt stress; FM, fresh matter; T, salt stress; D, ensiling day; T × D, the interaction between salt stress and ensiling days; SEM, standard error of means. Means with different letters in the same column (a–d) differ (p <0.05)*.

### Microbial Community of Alfalfa Silage

A high-throughput sequencing method was performed in variable regions 3 and 4 of 16s rDNA to calculate and evaluate bacterial diversity after ensiling (Table 5). Based on the coverage values, >99% of coverage values had adequately obtained most of the bacterial communities. The OTUs, Shannon, Ace, and Chao 1 values ranged from 44 to 83, 1.27 to 2.41, 69.78 to 99.12, and 66.58 to 100.13, respectively. In conclusion, salt stress greatly affected the microbiome of the resultant silage.

**Table 5:** Alpha diversity of the bacterial community in fresh materials and alfalfa silage.

**Treatment**	**Ensiling (days)**	**Sequence number**	**OTUs**	**Shannon**	**Ace**	**Chao 1**	**Coverage**
CK	0	16225	67	2.05	83.06	75.23	0.99
LS	0	18345	75	1.73	83.24	83.45	0.99
MS	0	20200	63	1.62	82.86	91.13	0.99
HS	0	13245	60	1.92	69.78	68.07	0.99
CK	30	17050	63	1.55	76.41	76.69	0.99
LS	30	20787	61	1.38	77.93	70.58	0.99
MS	30	22215	54	1.43	78.18	66.58	0.99
HS	30	18671	83	2.41	99.12	100.13	0.99
CK	60	17890	52	1.27	81.66	66.22	0.99
LS	60	23293	71	1.77	82.38	79.11	0.99
MS	60	18018	58	1.33	94.57	76.11	0.99
HS	60	14237	44	1.34	92.40	74.00	0.99

*CK, without salt stress; LS, under light salt stress; MS, under moderate salt stress; HS, under severe salt stress; OTUs, operational taxonomic units; fresh material of alfalfa; 30, 30 days of ensiling; 60, 60 days of ensiling*.

**Figure 1:**
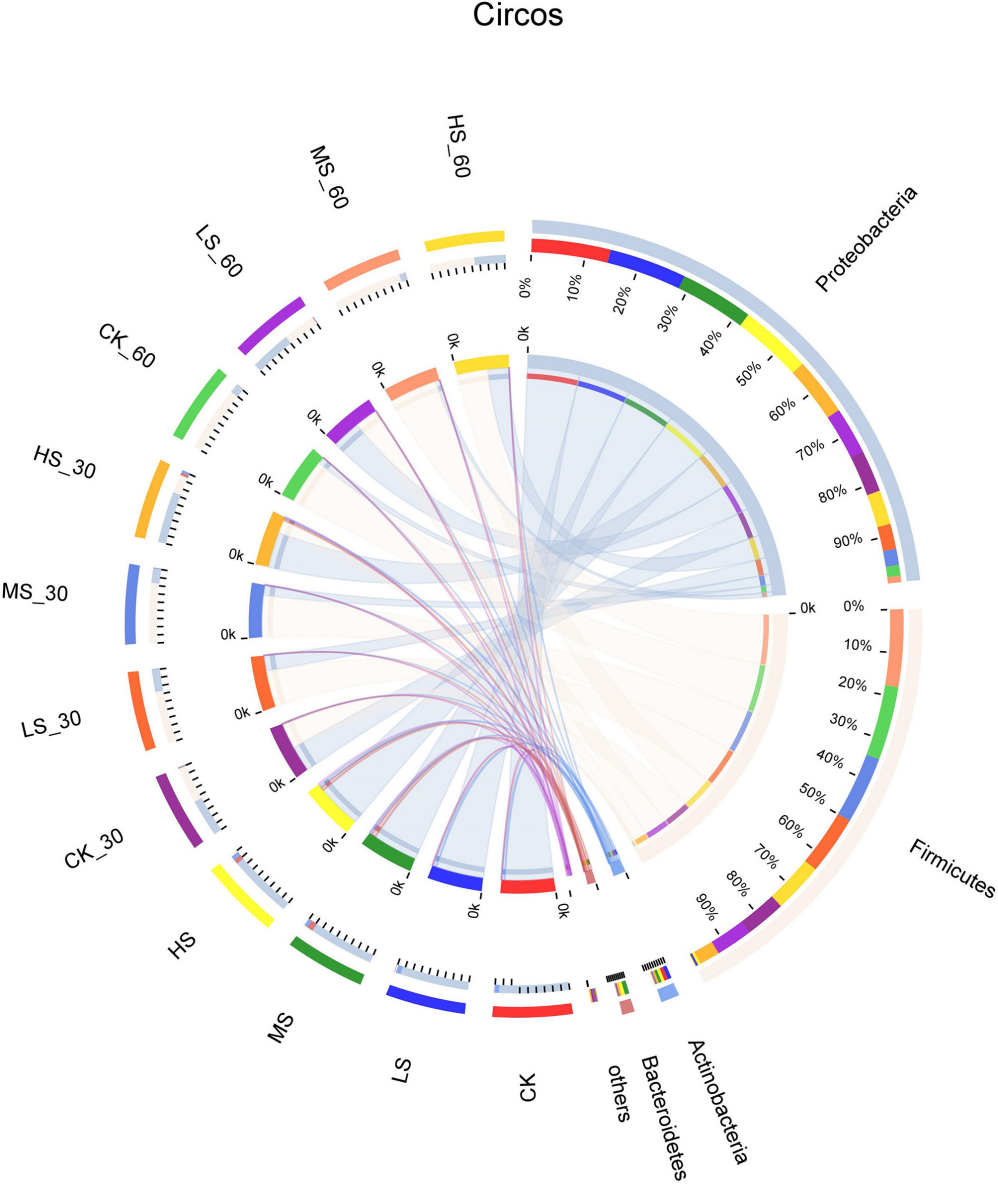
Differences in relative abundance of microbial community on phylum level shown by using a Circos plot. The size of the bars from each phylum shows the relative abundance of that phylum in the sample. CK, fresh material of alfalfa without salt stress; LS, alfalfa under light salt stress; MS, alfalfa under moderate salt stress; HS, alfalfa under severe salt stress; 30, 30 days of ensiling; 60, 60 days of ensiling.

Bacterial community dynamics at the phylum level during the ensiling of alfalfa are shown in Figure 1. The dominant phyla in CK microbiota were *Proteobacteria* (92.98%), *Actinobacteria* (5.9%), and *Firmicutes* (0.64%), whereas the LS microbiota was dominated by *Proteobacteria* (86.6%), *Actinobacteria* (4.47%), and *Firmicutes* (1.72%). The MS microbiota was dominated by *Proteobacteria* (96.5%), *Actinobacteria* (6.4%), and *Bacteroidetes* (8.27%). The HS microbiota was dominated by *Proteobacteria* (85.1%), *Actinobacteria* (5.3%), and *Bacteroidetes* (6.42%). The dominant phyla in fresh alfalfa microbiota were *Proteobacteria*. The proportions of *Firmicutes* increased while the relative abundance of *Proteobacteria* decreased with the extension of time.

**Figure 2:**
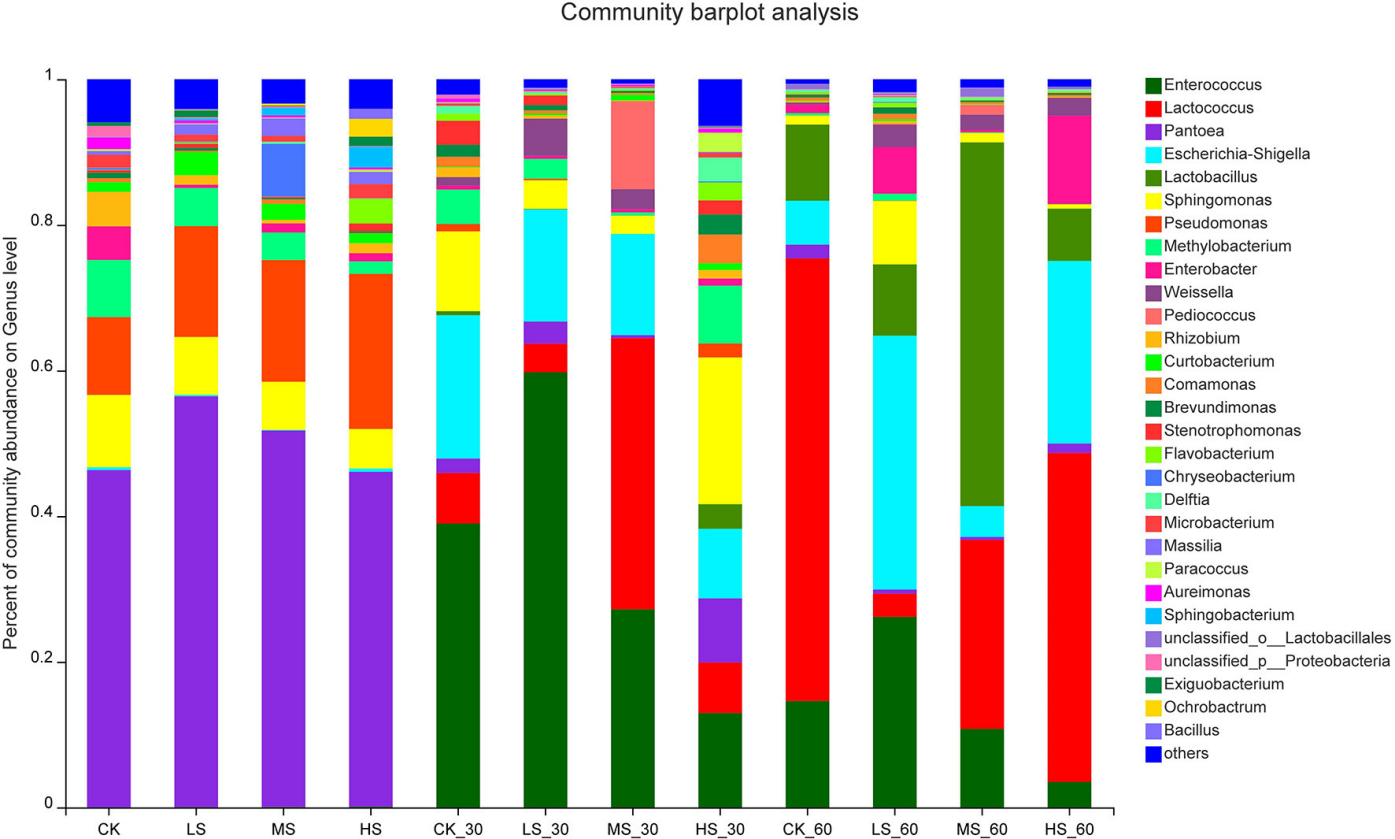
Bacterial community composition on genus level in alfalfa silage. CK, fresh material of alfalfa without salt stress; LS, alfalfa under light salt stress; MS, alfalfa under moderate salt stress; HS, alfalfa under severe salt stress; 30, 30 days of ensiling; 60, 60 days of ensiling.

**Figure 3:**
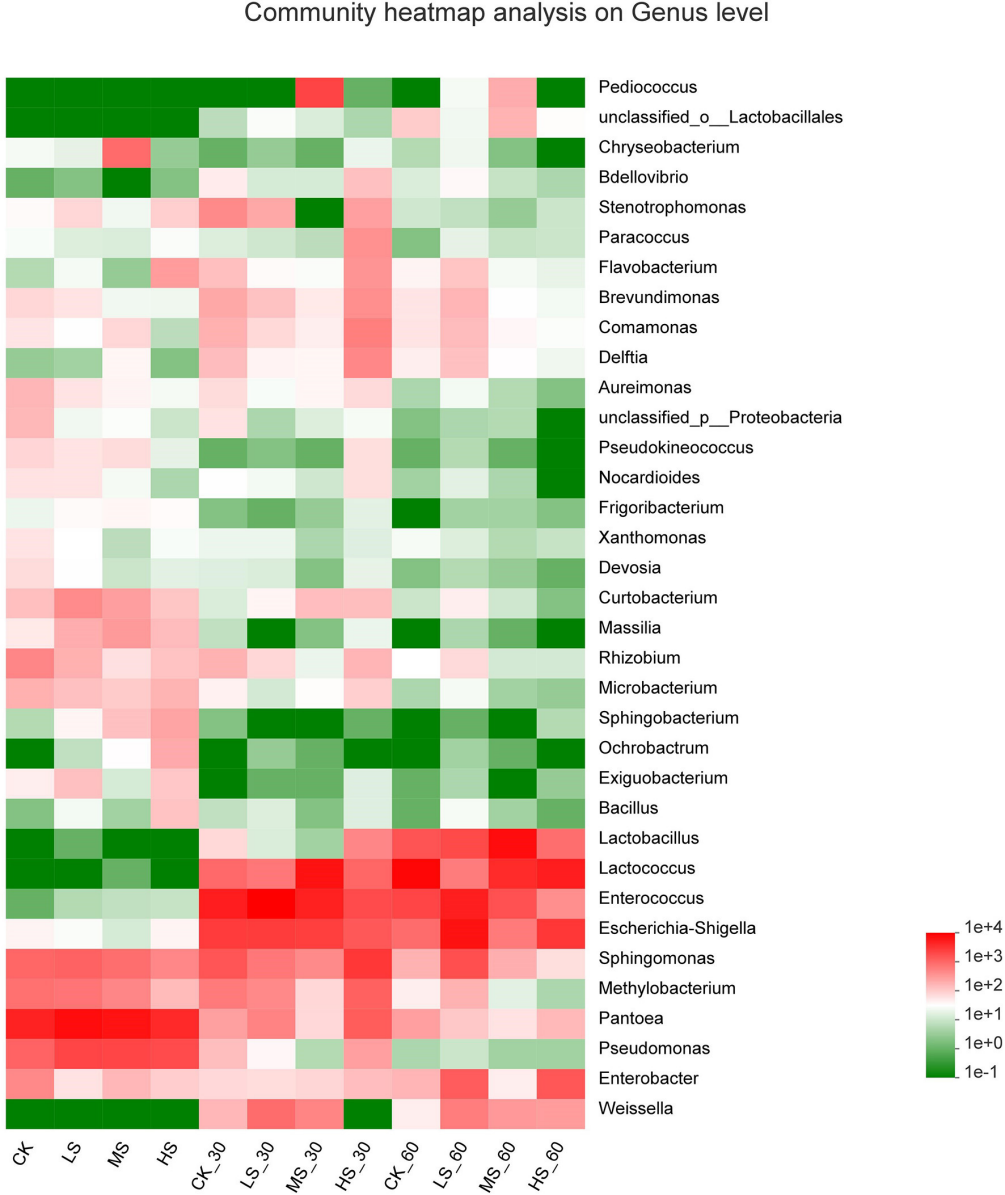
Heatmap of prominent bacterial genera (35 most abundant genera) for alfalfa silage. CK, fresh material of alfalfa without salt stress; LS, alfalfa under light salt stress; MS, alfalfa under moderate salt stress; HS, alfalfa under severe salt stress; 30, 30 days of ensiling; 60, 60 days of ensiling.

To further comprehend the succession of bacterial communities of alfalfa silage under salt stress, bacterial communities at the genus level were evaluated in Figures 2, 3. It shows that the alfalfa under salt stress used to produce the silage had a dramatic impact on the resultant microbial community. *Pantoea* were the dominant genera in CK (46.32%), LS (56.42%), MS (51.68%), and HS (45.98%); what is more, after 30 days and 60 days of ensiling, the relative abundance of *Pantoea* significantly decreased. After the fermentation process, *Enterococcus, Lactococcus*, and *Lactobacillus* were the dominant genera. Lower pH inhibited the growth and breeding of *Pantoea, Pseudomonas*, and *Methylobacterium*. Interestingly, with the increase of salt stress, the relative abundance of *Pantoea* decreased gradually, while that of *Pseudomonas* increased gradually. In the absence of oxygen, *Enterococcus* became the dominant genera in the CK_30 (38.99%) and LS_30 (59.75%). *Enterococcus* in CK_60 (14.6%) and LS_60 (26.17%) also decreased. *Sphingomonas* (20.15%) was found in HS_30. After 60 days of ensiling, *Lactococcus* became the dominant genera in the CK_60 and HS_60, *Lactobacillus* and *Lactococcus* became the dominant genera in the MS_60, and *Enterococcus* was still the dominant genera in the LS_60. With the decrease of pH, the acidic environment restricted the existence and relative abundance of *Enterococcus*, while the relative abundance of *Lactococcus* and *Lactobacillus* increased in the four groups.

**Figure 4:**
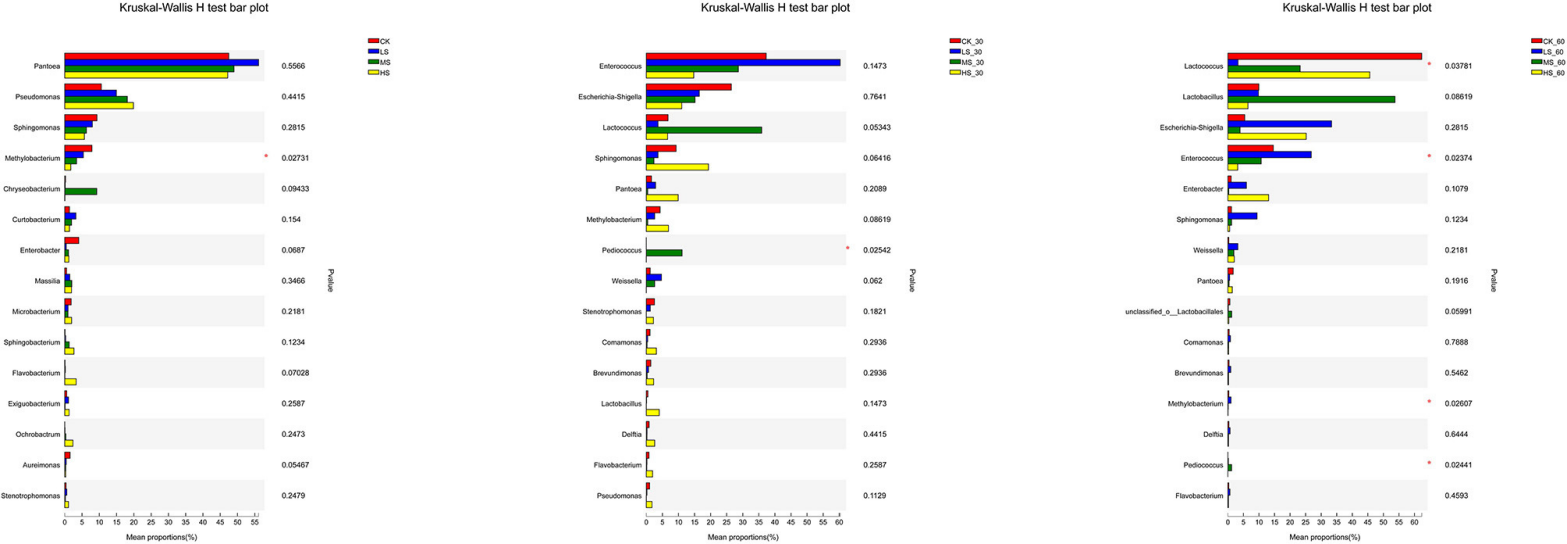
Comparison of microbial variations using the Kruskal–Wallis *H* test for alfalfa silage. *p < 0.05. CK, fresh material of alfalfa without salt stress; LS, alfalfa under light salt stress; MS, alfalfa under moderate salt stress; HS, alfalfa under severe salt stress; 30, 30 days of ensiling; 60, 60 days of ensiling.

To evaluate the bacterial communities of different silage groups under salt stress, the bacterial communities at the genus level were evaluated in Figure 4. With the gradual increase in salt stress, *Pseudomonas, Sphingomonas*, and *Methylobacterium* showed strong response patterns. In contrast, *Sphingomonas, Methylobacterium*, and *Aureimonas* decreased with increasing salt stress and showed salt-tolerant properties. After 30 days of ensiling, the abundance of *Weissella* and *Escherichia* would decrease with the increase of salt stress. Besides, the content of ammonia-N in MS_30 and HS_30 would be the lowest significantly (*p* < 0.05), and the same rules and patterns were also reflected in 60 days.

### The Relationships Among the Samples, Microbial Community, and Characteristics Products in Alfalfa Silage

**Figure 5:**
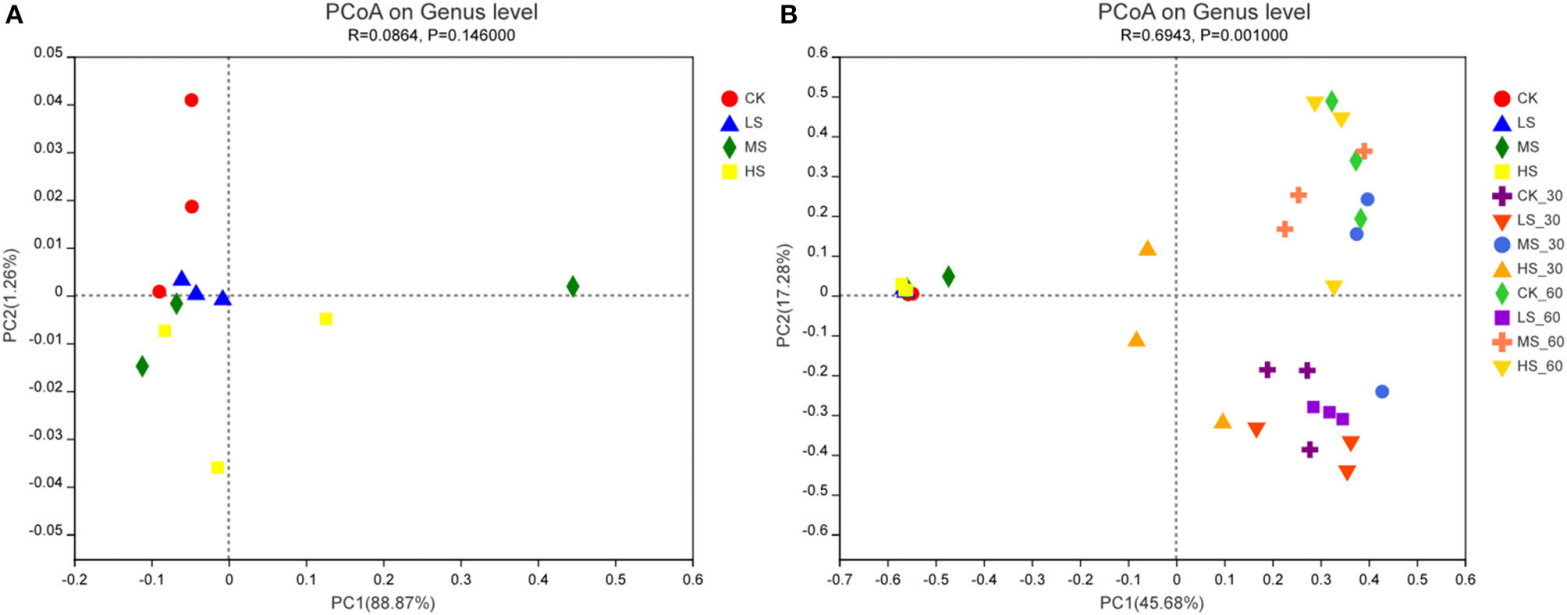
**(A,B)** Principal co-ordinates analysis (PCoA) of bacterial communities in alfalfa silage. CK, fresh material of alfalfa without salt stress; LS, alfalfa under light salt stress; MS, alfalfa under moderate salt stress; HS, alfalfa under severe salt stress; 30, 30 days of ensiling; 60, 60 days of ensiling.

The variations in the microbial communities of the silage were further verified by β-diversity analysis using principal coordinates analysis (PCoA) (Figure 5). The perspicuous separation among the microbiota indicated that different epiphytic microbial communities exist in alfalfa under different salt stress. As seen in Figure 5A, salt stress had a great influence on the microbial community of fresh plants. The results further validate the differences between the microbial communities in Figures 2, 3. By analyzing the composition of the microbial community structure, we would reveal the changes in microbial community structure under different salt stress. There were obvious differences in microbial community composition among different habitat types in Figure 5A. It is not easy to understand and master the distribution characteristics of microorganisms. Intriguingly, the microbial community structure was different at different fermentation stages, and small changes were found in LS_30 and LS_60, which might be due to the fact that *Lactococcus* and *Lactobacillus* were used as the dominant bacteria in the 60-day stage of the fermentation process.

**Figure 6:**
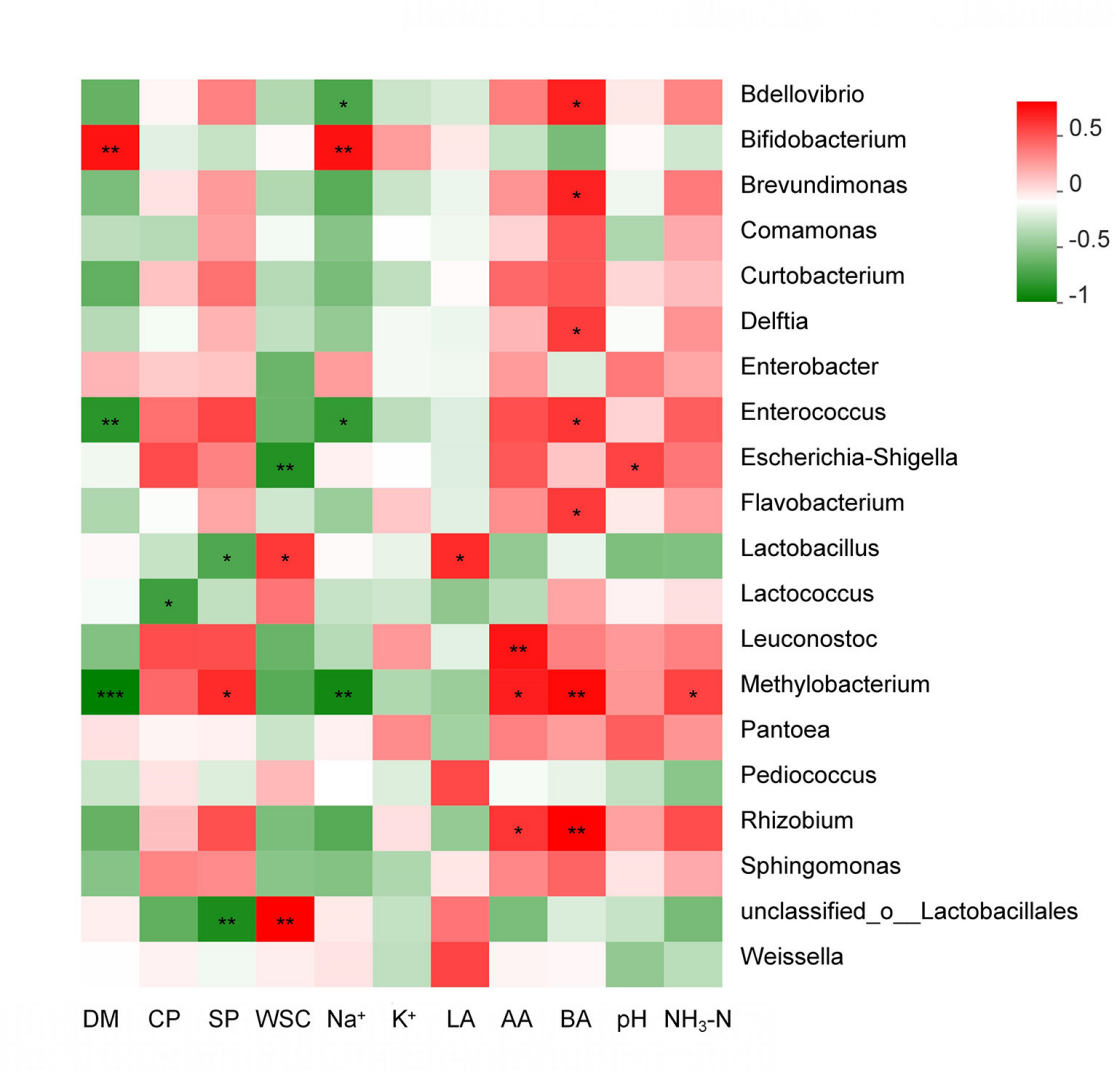
Relevance analysis of high-throughput sequencing data related to the genus level (20 most abundant); DM, dry matter; CP, crude protein; SP, soluble protein; LA, lactic acid; AA, acetic acid; BA, butyric acid; NH_3_-N, ammonia nitrogen; WSC, water-soluble carbohydrates. The legend on the right is the color interval of different *R* values. * 0.01 < *p* ≤ 0.05, ** 0.001 < *p* ≤ 0.01, *** *p* ≤ 0.001.

As seen in Figure 6, the obvious relationships among high-throughput sequencing data on the genus level and major fermentation products are visualized in a relevance analysis plot. Silage pH was positively correlated with the abundance of *Lactococcus, Escherichia*–*Shigella*, and *Pantoea* in the silage microbiome, while it was negatively correlated with the abundance of *Weissella*. The silage DM was negatively correlated with the abundance of *Enterococcus* and *Methylobacterium* (*p* < 0.01). The silage CP and SP were negatively correlated with the abundance of *Lactobacillales, Lactococcus*, and *Lactobacillus*. The silage WSC was negatively correlated with the abundance of *Escherichia*–*Shigella* (*p* < 0.01). The silage Na^+^ was positively correlated with the abundance of *Bifidobacterium* (*p* < 0.01), while it was negatively correlated with the abundance of *Bdellovibrio, Brevundimonas, Comamonas, Curtobacterium, Enterococcus, Flavobacterium, Methylobacterium*, and *Sphingomonas*. The silage LA was positively correlated with the abundance of *Lactobacillus* (*p* < 0.05). The silage BA was positively correlated with the abundance of *Enterococcus, Bdellovibrio, Brevundimonas, Delftia, Flavobacterium, Leuconostoc, Methylobacterium*, and *Rhizobium* (*p* < 0.05).

## Discussion

### The Chemical Composition of Fresh Alfalfa

Salt stress affects the normal growth and development of plants and affects the chemical composition of plants. Differences between SP in different groups can be considered as a detoxification effect of plants and there was a also significant difference in SP between LS and MS groups. SP is an important factor in relieving osmotic pressure in response to salt stress (Kanwal et al., 2019). The SP content in a low NaCl group increased significantly (Lacramioara et al., 2016); this is congruent with the findings of Agastian et al. (2000), who reported that the SP content in a high salinity growth environment was decreased (Agastian et al., 2000). The main storage form of energy in plants is non-structural carbohydrates (NSC), which include soluble carbohydrates (sucrose and fructose, etc.) and starch, which are the important sources of energy in the process of plant growth and metabolism. The WSC content (49 g/kg) in CK was significantly higher than in other groups, because salt stress inhibits the metabolism and accumulation of carbohydrates in plants (Morais et al., 2019). Salt stress is a likely factor to cause chemical composition changes. In the study, the colonies of LAB alfalfa provided enough bacteria to initiate the silage fermentation reported by Oliveira et al. (2017).

### Fermentation Characteristics and Chemical Composition of Alfalfa Silage

Silage pH is a very important evaluation indicator to consider when evaluating the fermentation efficacy; well-fermented silage should have a pH of 4.2 or lower (McDonald et al., 1991). In the study, the results presented that all silages had a pH of higher than 4.2 and showed a poor fermentation quality. The variety of pH might be caused by different alfalfa under salt stress, or differences in the silage microbiome. The differing chemical compositions of the alfalfa samples may have contributed to the substantial variation in silage fermentation characteristics; Xue et al. (2021) reported this phenomenon previously, albeit in *Pennisetum sinese* silage. The lower pH and high LA concentration of MS and HS silage were attributed to the LAB, which quickly metabolized WSC into LA, thus reducing silage pH and stability in a short time (Nazar et al., 2020). AA is derived from the decomposition and fermentation of sugars by heterofermentative LAB, Enterobacteriaceae, and Clostridium (Nazar et al., 2020).

In this study, there were *Weissella, Enterobacter*, and *Sphingobium* bacteria in the fermentation for 30 and 60 days. Weissella and Enterobacter could not only consume a large amount of WSC content but also have a low utilization rate of WSC (Blajman and Vinderola, 2020). It was also possible that with the increase of salt stress, more heterogeneous LAB was attached to alfalfa plants. BA and other acids reflect the efficiency of fermentation or secondary fermentation (Wang et al., 2020). Besides, the amount of BA also depends on the amount of LA; this may be caused by the secondary fermentation of heteromorphic LAB and yeast (Lei et al., 2015). The presence of ammonia-N in silage is an important indicator of the degree of proteolysis. Li et al. (2018) found that the process was mainly driven by plant protease activity and proteolytic Clostridia. It might be the reason that Na^+^ had impacted the activity of protease enzymes throughout the ensiling. When Na+ content is below 60 mg/L, Na^+^ could promote the activity of protease enzymes, while above 60 mg/L, it could inhibit the activity of protease enzymes instead. It might be that the NA^+^ content in MS and HS groups in this study inhibited the activity of protease enzymes.

From a perspective of the ammonia-N content of alfalfa silage, The ammonia-N is considered as a representation of proteolytic activity (Li et al., 2018). The inhibiting effect on ammonia-N accumulation of MS and HS suggested an enhancement in protein preservation during ensiling. A typical reason for ammonia-N accumulation was the proteolytic enzymes (Kung and Shaver, 2001). Even most plant proteolytic enzymes in alfalfa silage showed greater activities at pH 5.0–6.0 (Yang et al., 2019), but excess ions can inhibit proteolytic enzymes (Jaci et al., 2014), which might be an explanation for the reduction in ammonia-N formation in the MS and HS silage. After alfalfa fermentation, the content of SP increased from 66–108 to 124–150, which may be related to the hydrolysis of protein during the fermentation process. The protein of macromolecules was decomposed into small molecular proteins with water-soluble characteristics (Liu et al., 2021), which might be an explanation for the increase of SP.

### Microbial Community of Alfalfa Silage

In the present study, *Firmicutes, Bacteroidetes, Proteobacteria*, and *Actinobacteria* were dominant in all alfalfa silage. These findings are consistent with previous reports examining alfalfa silage and even corn silage (Lei et al., 2015; Blajman and Vinderola, 2020). *Firmicutes* and *Proteobacteria* were the most abundant phyla in the fermentation silage (Jie et al., 2020). The transformation of the bacterial community from *Proteobacteria* to *Firmicutes* was reported by Nazar et al. (2020), who revealed that the anaerobic and acidic environment quite sustained the growth of *Firmicutes*. Besides, with the increase of salt stress, the relative abundance of *Proteobacteria* decreased regularly. Luttenton and Rada, 2010 had stated that the same plants have different epiphytes flora as if they grow under different environmental conditions; salt stress could be the reason for the changes of microbial communities in this study. The response of plant microorganisms to different salt stresses was very interesting and beyond expectation. With the gradual increase in salt stress, *Pseudomonas, Sphingomonas*, and *Methylobacterium* showed strong response patterns. *Pseudomonas* increased with salt stress increased, which may be since these two genera prefer to survive and reproduce in salinity environments (Nidhi et al., 2013).

Compared to raw materials, the anaerobic environment causes the changes of microbial habitat, and the silage process inhibited the aerobic microorganisms, which contributed to the differences in microbial communities of the fresh alfalfa and silages (Nazar et al., 2020); the diversity and richness of bacteria decreased in CK, LS, MS, and HS silages, whether in 30 or 60 days. Similarly, Jie et al. (2020) reported that silage bacterial diversity decreased due to the increase in the abundance of the predominant genus (*Lactococcus* and *Enterococcus*). When the dominant bacteria were abundant, the microbial community of the silage would be decreased (Nazar et al., 2020). With the increase of salt stress, the bacteria diversity and richness of raw alfalfa were decreased and then increased. This phenomenon could be caused by competition between bacteria due to antibacterial activity. Heinz et al. (2019) revealed a hypothesis that halophilic bacteria like *Planococcus halocryophilus* manifested different phenotypic responses under high salt stress (>8%Nacl). *Planococcus halocryophilus* can develop perchlorate-specific stress adaptations that were not (or only to a lower extent) used to counteract high Na^+^ concentrations.

After the fermentation process, *Enterococcus, Lactococcus*, and *Lactobacillus* were the dominant genera. Lower pH inhibited the growth and breeding of *Pantoea, Pseudomonas*, and *Methylobacterium*. Interestingly, with the increase of salt stress, the relative abundance of *Pantoea* decreased gradually, while that of *Pseudomonas* increased gradually. It was reported that *Pantoea* and *Pseudomonas*, as PGPR (plant-growing-promoting Rhizobacteria), could produce IAA and promote biomass to help plants survive better under salt stress (Sun et al., 2020), but *Pantoea*'s adaptation to the halophytic environment was limited, so its abundance would gradually decrease, while *Pseudomonas* was more adaptive to the halophytic environment than *Pantoea* (Ching and Deivanai, 2015). In the absence of oxygen, *Enterococcus* became the dominant genera in the CK_30 (38.99%) and LS_30 (59.75%). *Enterococcus* was often used to enhance the fermentation characteristics reported by Wang et al. (2020). *Enterococcus* could rapidly produce LA and establish an acidic anaerobic environment to promote LAB growth in the early fermentation stage, but *Enterococcus* is not an acid-resistant genera (Cai et al., 1998). Similarly, the same conclusion was also verified in this study; *Enterococcus* in CK_60 and LS_60 also decreased significantly. A large abundance of *Sphingomonas* has existed in HS_30. *Sphingobium* could cause further hydrolysis of CP and SP (Zhou et al., 2019). *Sphingobium* might cause an increase in ammonia-N.

*Lactococcus* generally existed in naturally fermented silages. This was not consistent with the conclusion of Wang et al. (2020), possibly because no additives were used in this study and only rely on the microorganisms attached to alfalfa under salt stress. It is undeniable that *Lactobacillus* and *Lactococcus* are important in pH decline and LA accumulation, and in the whole process of silage, they are generally present. Wang et al. (2020) stated that the composition of microorganisms in silage was quite different at the initial stage of the fermentation. *Lactobacillus* could grow vigorously at the end of the fermentation stage due to their stronger acid resistance than cocci LAB. *Lactobacillus* could become the dominant bacteria at the end of the fermentation stage because of its stronger acid resistance than *Lactococcus*. Generally, LA in silage is mainly produced by *Lactobacillus*, while with the decrease of pH, *Lactococcus* and *Enterococcus* could not survive with lower pH due to weaker acid resistance.

This study found that there was a significant correlation between the chemical composition of silage and silage microorganisms. The chemical composition and microbial community of alfalfa under different salt stress were different, so the characteristics of the silage microbial community were also different. This may be explained by the fact that these microorganisms are chemoorganotrophic bacteria that produce energy through the oxidation of organic matter such as starch and organic acids. *Pseudomonas, Bacteroides*, and *Stenotrophomonas* all consume protein, while *Megamonas, Raoultella, Citrobacter*, and *Enterococcus* ferment carbohydrates (Tian et al., 2019; Xue et al., 2021). The results of this study further compound the significant relationship between the chemical composition of the raw material and the resultant silage.

Intriguingly, Na^+^ and K^+^ would inhibit the growth and reproduction of most microorganisms; although ions are not directly responsible for the growth of microorganisms, bacteria can be able to look at potassium ion channel–electrical signaling to achieve cell–cell communication (Jing et al., 2020). This could explain the fact that the number and diversity of microbes were low under salt stress (Table 5). Ion stress inhibited not only the activity and abundance of microorganisms but also the breeding of harmful microorganisms. The composition and distribution of microorganisms could be clearly defined, and it would be of great significance to the improvement and development of ecology and biogeography as well as the protection and utilization of microbial resources.

## Conclusion

The study presented herein examined the microbiome and quality of silages from the natural fermentation of alfalfa under salt stress. Salt stress has a great impact on the quality and bacterial community of fresh alfalfa and silage. There was a predominance of desirable genera including *Lactococcus* and *Lactobacillus* in silage produced from alfalfa under salt stress, and this led to better fermentation quality. There was a strong correlation between the chemical composition and bacterial community of the silage. The salt stress and plant ions were thus most responsible for their different fermentation modes in alfalfa silage. The results of the study indicate that exogenous epiphytic microbiota of alfalfa under salt stress could be used as a potential bioresource to improve the fermentation quality.

## Data Availability Statement

The datasets presented in this study can be found in online repositories. The names of the repository/repositories and accession number(s) can be found here: NCBI PRJNA753242.

## Author Contributions

QL: writing original draft. DS and ZJW: writing review and editing. GG and ZW: data curation. YJ: methodology. MH: software. All authors have read and agreed to the published version of the manuscript.

## Funding

This study was funded by the Inner Mongolia Autonomous Region Science and Technology Project (201802069), the National Natural Science Foundation of China (42077054), and the National Technical System of Forage Industry for Dry Grass Storage (CARS-34).

## Conflict of Interest

The authors declare that the research was conducted in the absence of any commercial or financial relationships that could be construed as a potential conflict of interest.

## Publisher's Note

All claims expressed in this article are solely those of the authors and do not necessarily represent those of their affiliated organizations, or those of the publisher, the editors and the reviewers. Any product that may be evaluated in this article, or claim that may be made by its manufacturer, is not guaranteed or endorsed by the publisher.
