# Supplementary figures and images for: The Potential Effects on Microbiota and Silage Fermentation of Alfalfa Under Salt Stress
Source: Front Microbiol. 2021 Oct 11;12:688695. doi: 10.3389/fmicb.2021.688695 (PMC8544858; doi:10.3389/fmicb.2021.688695)

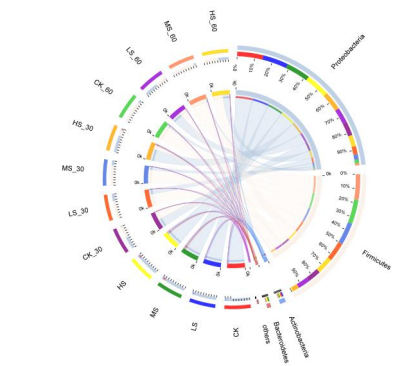

# Epiphytic microbiota

Alfalfa      Salt stress

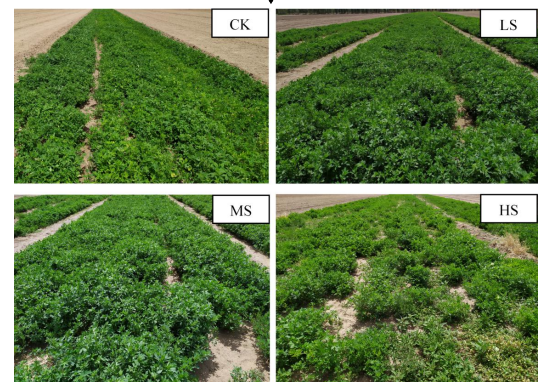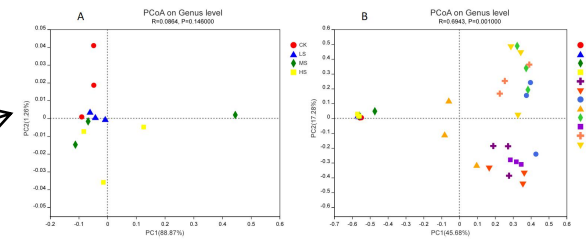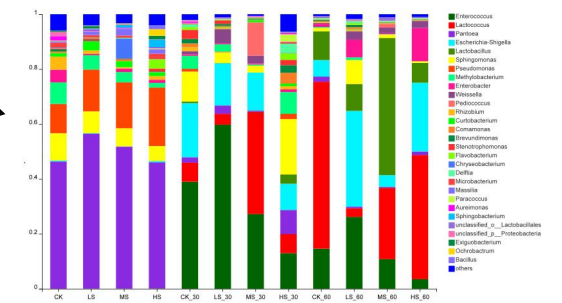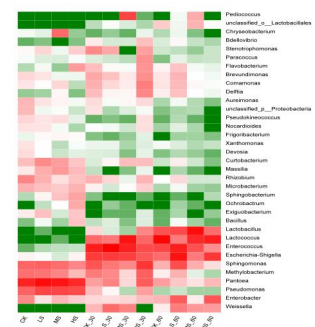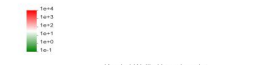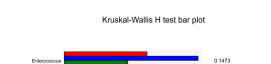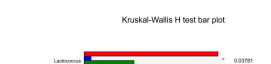

Supplement: Supplementary file 2 [file Image_1.PDF]
